# The Natural History of Man; Comprising Inquiries into the Modifying Influence of Physical and Moral Agencies on the Different Tribes of the Human Family

**Published:** 1843-01

**Authors:** 


### ART. XV.

*The Natural History of Man; comprising Inquiries into the Modifying Influence of Physical and Moral Agencies on the different Tribes of the Human Family.* By JAMES COWLES PRICHARD, M.D. F.R.S. M.R.I.A. &c.—*London*, 1843. 8vo, pp. 556.

ON a former occasion, (Br. & For. Med. Review, vol. XIII. p. 521,) we noticed this work with due commendation on the appearance of its early numbers. It is now completed, and forms one of the handsomest as well as most interesting volumes that have appeared for a long time. It has the rare merit of being equally suited to the well-informed general reader and to the medical professor; and we doubt not but it will attain, what it so richly merits, universal attention and approbation from the public. As we said on a former occasion, the work is worthy its distinguished author and of his former works, and we can give it no higher praise.

Our limited space forbids our entering at all upon an analysis of the work, but we must give our readers a general notion of its scope and objects, and how these are worked out by the author; and this we shall do by a few brief extracts from its own pages.

*Object of the work.* "It is the design of the following work, to furnish for the use of general readers, a brief and popular view of all the physical characteristics, or varieties in colour, figure, structure of body, and likewise of the moral and intellectual peculiarities which distinguish from each other the different races of men. It is likwise intended, in the same treatise, to comprise such an account of the nature and causes of these phenomena as the present state of knowledge will afford." (p. vii.)

*Bearings of the question.* "The sacred scriptures, whose testimony is received by all men of unclouded minds with implicit and reverential assent, declare that it pleased the Almighty Creator to make of one blood all the nations of the earth, and that all mankind are the offspring of common parents. But there are writers in the present day who maintain that this assertion does not comprehend the uncivilized inhabitants of remote regions; and that Negroes, Hottentots, Esquimaux, and Australians are not, in fact, men in the full sense of that term, or beings endowed with like mental faculties as ourselves. Some of these writers contend that the races above mentioned, and other rude and barbarous tribes, are inferior in their original endowments to the human family which supplied Europe and Asia with inhabitants—that they are organically different, and can never be raised to an equality, in moral and intellectual powers, with the offspring of that race which displays in the highest degree all the attributes of humanity." (p. 5.)

The first step in the inquiry is an examination of the import of the terms *genus*, *species*, and *variety*, and the conclusion arrived at is briefly stated as follows:

"It seems to be the well-established result of inquiries into the various tribes of organized beings, that the perpetuation of hybrids, whether of plants or of animals, so as to produce new and intermediate tribes, is impossible. Now, unless all these observations are erroneous, or capable of some explanation that has not yet been pointed out, they lead, with the strongest force of analogical reasoning, to the conclusion that a number of different tribes, such as the various races of men, must either be incapable of intermixing their stock, and thus always fated to remain separate from each other, or, if the contrary should be the fact, that all the races to whom the remark applies are proved by it to belong to the same species." (pp. 17-18.)

The next stage of the inquiry comprehends a survey of the variations or degenerations undergone by plants and animals from climate, domestication, and other circumstances, and the result is given as follows:

"1. That tribes of animals which have been domesticated by man and carried into regions where the climates are different from those of their native abode, undergo, partly from the agency of climate, and in part from the change of external circumstances connected with the state of domesticity, great variations.

"2. That these variations extend to considerable modifications in external properties, colour, the nature of the integument, and of its covering, whether hair or wool; the structure of limbs, and the proportional size of parts; that they likewise involve certain physiological changes or variations as to the laws of the animal economy; and lastly, certain psychological alterations or changes in the instincts, habits, and powers of perception and intellect.

"3. That these last changes are in some cases brought about by training, and that the progeny acquires an aptitude to certain habits which the parents have been taught; that psychical characters, such as new instincts, are developed in breeds by cultivation.

"4. That these varieties are sometimes permanently fixed in the breed so long as it remains unmixed.

"5. That all such variations are possible only to a limited extent, and always with the preservation of a particular type, which is that of the species. Each species has a definite or definable character, comprising certain undeviating phenomena of external structure, and likewise constant and unchangeable characteristics in the laws of its animal economy and in its psychological nature. It is only within these limits that deviations are produced by external circumstances." (pp. 74-5.)

The application of these conclusions to the case of the human animal is obvious:

"Races of men are subjected more than almost any race of animals to the varied agencies of climate. Civilization produces even greater changes in their condition than does domestication in the inferior tribes. We may therefore expect to find fully as great diversities in the races of men as in any of the domesticated breeds. The influence of the mind must be more extensive and powerful in its operations upon human beings than upon brutes. And this difference transcends all analogy or comparison. *A priori*, we might expect to discover in the psychological characters of human races changes similar in kind, but infinitely greater in degree." (p. 75.)

To test this *à priori* argument by facts as they exist, the author enters upon a most elaborate and complete examination, physical, moral, and intellectual, of all the nations, tribes, and races of men found throughout the globe. To this survey the greater part of the volume is devoted. It is conducted with astonishing learning, admirable judgment, and in the most impartial and philosophical spirit. The following extracts contain, in a few words, an outline of the principal conclusions arrived at by the author:

1. *Physical or anatomical identity.* "The different races of men are not distinguished from each other by strongly marked, uniform, and permanent distinctions, as are the several species belonging to any given tribe of animals. All the diversities which exist are variable, and pass into each other by insensible gradations; and there is, moreover, scarcely an instance in which the actual transition cannot be proved to have taken place. Thus, if we consider the varieties of figure which are generally looked upon as the most important, and begin with those of the skeleton and the skull as their foundation, we shall find every particular type undergoing deviations and psssing into other forms. We have seen that, in many races who have, generally and originally, as far as we can go back towards their origin, heads of the pyramidal figure with broad faces, or the Mongolian type, the oval or European shape with European features display themselves in individuals, and often become the characteristics of tribes.

"Again, the shape of the head in the black races varies in like manner. The Sudanian nations have a black complexion and crisp hair, with a form of the head different from that of the negro; and the type varies in particular tribes, and even in the same tribe. Towards the south, the black and crisp-haired Africans display, in the highland of the Kafirs, a form resembling the European; and, in the country of the nomadic Hottentots, make a signal approximation to the physical character prevalent among the nomades of High Asia. Among the aboriginal races of the New World, similar varieties and similar deviations occur.

"With respect to colour, it is still more easy to trace the greatest variations within the limits of one race. There is, perhaps, not one great family of nations, having its branches spread through different climates, which does not display in this particular the most strongly marked varieties. It is true that among European colonists settled in hot climates such varieties are not so perceptible within a few generations; but in many well-known instances of earlier colonization they are very clearly manifested. We have traced them in the instances of the Jews and Arabs, in the tribes of Hindoos, or rather of the Indian race, spread through India, compared with those of the Himâlayan countries. We might add innumerable facts tending to bring out the same result.

"It has often been said that the native tribes of America present an exception to the general observation deduced from a survey of the nations of the Old World, and that the complexion of the Americans displays no relation to climate. We have proved, on the contrary, that tribes alike belonging to the American stock manifest the influence of external agencies not less distinctly than do the white inhabitants of Europe compared with the black races of Africa. Witness the comparison of the white Americans of the north-west coast with the black Californians.

"If any one should call in question the assertion that the colour of human races has any relation to the climates of different countries, we have only need to appeal to the most general and broadly marked facts which the history of mankind presents. Thus it is obvious that the intertropical region of the earth is the principal seat of the black races of men, and the region remote from the tropics that of the white races, and that the climates approaching to the tropics are generally inhabited by nations which are neither of the darkest nor of the fairest complexion, but of an intermediate one. To this observation it may be added that high mountains and countries of great elevation are generally inhabited by people of lighter colour than those where the level is low, such as sandy or swampy plains on the sea-coast. Thus, if we begin with Africa, we shall find a great number of distinct races, as far as a total diversity of languages can be thought to distinguish men into separate races, spread over that great continent; and it may be observed that those whose abode is between the tropics, though differing from each other in many peculiarities, agree in the darkness of their complexion. In fact, if we divide Africa into three portions, we may define by the tropics the extent of the black complexion in its inhabitants. The nature of the hair is, perhaps, one of the most permanent characteristics of different races. The hair of the negro has been termed woolly: it is not wool, and only differs from the hair of other races in less important respects.

"The texture of the hair affords in the animal kingdom no specific characters. In mankind we find it in every gradation of variety; and if we take the African nations, I mean the black tribes who are apparently of genuine native origin, as one body, we shall discover among them every possible gradation in the texture of the hair, from the short close curls of the Kafir to the crisp but bushy locks of the Berberine, and again, to the flowing hair of the black Tuaryk or Tibbo. In some instances, indeed, it appears that the change from one to the other may be shown in actual transition." (pp. 473-6.)

2. *Physiological identity.* "The difference of climate occasions very little, if any, important diversity as to the periods of life and the physical changes to which the human constitution is subject; and that in all these great regulations of the animal economy, if we may use such an expression, mankind, whether white or black, are placed by nature nearly on an equal footing. As the duration of life and the age of adult growth is known to be nearly the same, it would be contrary to all probability should any material difference be found to prevail in respect to any one particular function or set of functions." (p. 486.)

3. *Psychical and moral identity.* "We contemplate among all the diversified tribes, who are endowed with reason and speech, the same internal feelings, appetencies, aversions; the same inward convictions, the same sentiments of subjection to invisible powers, and, more or less fully developed, of accountableness or responsibility to unseen avengers of wrong and agents of retributive justice, from whose tribunal men cannot even by death escape. We find every where the same susceptibility, though not always in the same degree of forwardness or ripeness of improvement, of admitting the cultivation of these universal endowments, of opening the eyes of the mind to the more clear and luminous views which Christianity unfolds, of becoming moulded to the institutions of religion and civilized life: in a word, the same inward and mental nature is to be recognized in all the races of men. When we compare this fact with the observations which have been heretofore fully established as to the specific instincts and separate psychical endowments of all the distinct tribes of sentient beings in the universe, we are entitled to draw confidently the conclusion, that ALL HUMAN RACES ARE OF ONE SPECIES AND ONE FAMILY." (pp. 545-6.)

It would be injustice to the author as well as the publisher if we did not call attention to the admirable manner in which this work is illustrated. It contains no less than "thirty-six coloured and four plain illustrations engraved on steel, and ninety engravings on wood." The greater part of these illustrations are portraits of individuals of every variety of the human family, especially of the more uncivilized tribes. Looking at these and at the way in which the whole work is got up, we must in justice withdraw the charge we formerly made of its being too-high priced. The first numbers gave us no reason to expect that the illustrations would be so numerous and beautiful as they really are.

